# National Guidelines for the Performance of the Sweat Test in Diagnosis of Cystic Fibrosis on behalf of the Croatian Society of Medical Biochemistry and Laboratory Medicine and the Cystic Fibrosis Centre - Paediatrics and adults, University Hospital Centre Zagreb

**DOI:** 10.11613/BM.2022.010501

**Published:** 2022-02-15

**Authors:** Jasna Leniček Krleža, Merica Aralica, Duška Tješić-Drinković, Karolina Crneković, Jelena Culej, Gordana Fressl Juroš, Verica Horvat, Dara Metzner, Dijana Pamuković Jaram, Alma Pipić Kitter, Fran Smaić, Sanela Šimić Vojak, Livija Šimičević, Valentina Verić

**Affiliations:** 1 Department of Laboratory Diagnostics, Children’s Hospital Zagreb, Zagreb, Croatia; 2 Clinical Department of Laboratory Diagnostics, Clinical Hospital Center Rijeka, Rijeka, Croatia; 3 Cystic Fibrosis Centre - Paediatrics and Adults, University Hospital Centre Zagreb and University of Zagreb - School of Medicine, Zagreb, Croatia; 4 Department of Medical Laboratory Diagnostics, General Hospital „Dr. Ivo Pedišić“, Sisak, Croatia; 5 Poliklinika Breyer, Zagreb, Croatia; 6 Department of Clinical Laboratory Diagnostics, Children’s Hospital Srebrnjak, Zagreb, Croatia; 7 Department of Medical Laboratory Diagnostics, General Hospital Varaždin, Varaždin, Croatia; 8 Department of Medical Laboratory Diagnostics, General Hospital Šibenik, Šibenik, Croatia; 9 Department of Medical Laboratory Diagnostics, General Hospital „Dr. Josip Benčević“, Slavnoski Brod, Croatia; 10 Department of Medical Laboratory Diagnostics, County Hospital Požega, Požega, Croatia; 11 Cinical Department of Laboratory Diagnostics, University Hospital Centre Zagreb, Zagreb, Croatia; 12 Department of Medical Laboratory Diagnostics, General Hospital Bjelovar, Bjelovar, Croatia

## Abstract

The sweat test (ST) is a cornerstone in the diagnosis of cystic fibrosis (CF), together with newborn screening and genetic testing. However, the performance of the ST can depend on the operator’s skill, so several international guidelines have been published to standardise the ST, but inconsistencies remain. The joint Working Group for ST Standardisation (WG STS) of the Croatian Society of Medical Biochemistry and Laboratory Medicine, in association with cistic fybrosis health professional and the Cistic Fibrosis Centre for Paediatrics and Adults, have issued National Guidelines for the Performance of the Sweat Test in order to ensure consistency in ST performance and accuracy of reported results. Many of the standards were taken from the 2nd Edition of the UK Guidelines for Performance of the ST for the Diagnosis of CF, while others were taken from independent consensus statements from the WG STS based on local ST equipment and practices. The standards cover every step of the ST, from the indications for testing to reporting of results and their interpretation, including the analytical phase and quality control. In addition, National Guidelines include appendices with practical examples in order to aid implementation of the recommendations in routine practice.

## Introduction

### Cystic fibrosis

Cystic fibrosis (CF) is an autosomal recessive disease caused by two mutations in the gene encoding the cystic fibrosis transmembrane conductance regulator (CFTR) protein which is found in the membranes of most epithelial cells in the human body (*1*).

CFTR protein primarily regulates the transport of electrolytes through the cell membrane. Its widespread expression in different tissues explains the multisystemic nature of CF. CFTR dysfunction leads the fluid produced by organs to become viscous and to accumulate, so CF frequently presents as chronic lung disease, malabsorption followed by malnutrition, male infertility, and salt wasting syndrome, with the most common symptom of CF being excessive excretion of salt in sweat (*2*). Over time, patients with CF develop complications such as diabetes, low bone mineral density and chronic liver disease (*3*). Although CF is a monogenic disease, its clinical presentation varies from patient to patient, even among those with the same genotype, and the symptoms of one individual can vary during the course of the disease (*1*).

Cystic fibrosis is considered the most common hereditary disease in Caucasians, with a worldwide incidence of 0.25-5 per 10,000 live births (*4*). The prevalence of the disease in the European Union is 0.75 per 10,000, which makes CF a rare disease (*5*). However, the total number of patients with CF is growing, reflecting improvements in diagnosis and treatment, so the number of affected adults is expected to substantially exceed the number of affected children in the future (*6*). According to published data from the European Cystic Fibrosis Society (ECFS) Patient Registry, 49,886 CF patients were registered in Europe in 2018, of whom 51.2% were adults (*7*). Croatia is included in the ECFS Patient Registry *via* its Database of Cystic Fibrosis Patients, which contained 132 patients in 2018, of whom 37% were adults. Demographic and epidemiological data suggest that 12-14 newborns each year have CF in Croatia (*8*, *9*). There is an international consensus on the criteria for diagnosing CF. The first guideline was published in 1998, and the Croatian Society of Paediatric Gastroenterology, Hepatology and Nutrition (CSPGHN) of the Croatian Medical Association (CMA) published “A protocol for the Diagnosis of Cystic Fibrosis” in 2004 (*2*, *10*). Over the years, international guidelines have been updated in accordance with new findings, with the

Consensus Guidelines from the Cystic Fibrosis Foundation (published in 2017) considered the current standard (*11*, *12*). Throughout their evolution, guidelines have retained the same basic starting points: in a patient showing clinical signs of the disease, impaired CFTR protein function should be tested to confirm the diagnosis. Clinical suspicion means that a person has at least one of the characteristic symptoms, tested positive during newborn screening for CF (not yet available in Croatia), or has a close family member with CF (*13*). One of the indicators of impaired CFTR protein function is elevated chloride concentration in sweat, as measured using the sweat test (ST) (*12*). The ST is therefore the first and most important laboratory test to confirm a CF diagnosis. Two other tests can assess CFTR protein function, but they are not widely used: *in vivo* measurement of the potential difference across the nasal mucosa, and *ex vivo* intestinal current measurement on rectal biopsies. Instead of demonstrating impaired CFTR protein function, diagnosis can also be confirmed by genetic analysis, but only by identifying two mutations that undoubtedly cause CF (*2*). More than half of patients newly diagnosed with CF in many European countries and around the world are diagnosed due to the newborn screening (*12*). Some infants that score positive during newborn screening but otherwise fail to fulfill other diagnostic criteria are described as having „CFTR-related metabolic syndrome (CRMS)” or „CF screen positive - inconclusive diagnosis (CFSPID)” (*14*). Since newborn screening is not performed in Croatia, the diagnosis of CF depends solely on the clinician’s awareness of disease symptoms from the neonatal period through adulthood. Regardless of whether screening is implemented or not, and regardless of how extensively genetic analysis is performed, the ST is the main test that reliably confirms disease. Therefore, the ST must be performed correctly and its results interpreted accurately for reliable confirmation of CF diagnosis (*12*).

### Sweat test

The ST includes a series of procedures to evaluate CFTR protein function in the sweat glands of children and adults: stimulating sweating, collecting the sweat, measuring chloride concentration, and reporting and interpreting results. In CF patients, impaired CFTR protein function leads to elevated chloride concentration in sweat, which has given rise to an informal name of the ST in Croatian as „sweat chloride concentration measurement”.

The ST is considered useful for diagnosing CF in 95% of patients. Recent studies show chloride concentration in sweat to be an outcome measure in clinical trials involving CFTR modulators (*15*, *16*).

The preanalytical phase of the ST includes patient preparation, stimulation of sweating and sweat collection by pilocarpine iontophoresis (PI). The sweat glands are stimulated by applying weak current and a solution of pilocarpine nitrate soaked onto filter paper or gauze, or applied as a gel onto the subject’s forearm. Then sweat is collected onto chloride-free filter paper or gauze, or into a commercial capillary tube system. PI may be performed using a commercial system or the original or modified method of Gibson and Cooke (*17*). Before commercial PI systems became available, laboratories or paediatric outpatient clinics would typically use in-house equipment and materials adapted for the Gibson and Cooke method (*18*, *19*).

The analytical phase of the ST includes chloride concentration measurement in the collected sweat using a validated method or conductivity measurement of sweat anions (chlorides, bicarbonates, lactates). Regardless of the analytical method, ST results are expressed in mmol/L. With the conductivity method, the result includes the concentration not only of chloride but also of other sweat anions, so the value represents the molar concentration of pure sodium chloride (NaCl) solution that has the same conductivity as the sweat sample at the same temperature; thus, the result is expressed as NaCl equivalents in mmol/L (*20*). According to current guidelines, the conductivity method can be used for screening, but not as a reference method for CF diagnosis (*12*, *21*).

Commercial systems are available at the market for the pre-analytical and analytical phases separately or combined into an all-in-one system like the Nanoduct Neonatal Sweat Analysis System (Nanoduct, ELITechGroup, USA). It has begun to be used at some healthcare institutions in Croatia. It uses PI to stimulate sweating and it relies on the conductivity method to measure anions in sweat (*22*).

In the postanalytical phase, interpretation of the ST results depends on the indication for testing. The indication may be to confirm or exclude a CF diagnosis or to monitor the response of patients receiving CFTR modulator therapy. A flowchart of the ST is shown in Appendix 1.

The aim of the National Guidelines for the Performance of the Sweat Test (NGPST) in the diagnosis of CF is to standardise the ST across healthcare centres in Croatia, ensuring consistent procedural quality as well as accuracy of the results and their interpretation.

## Guidelines for the performance of the sweat test

### International guidelines for the performance of the sweat test

Several national guidelines for performing the ST have been published in Europe and worldwide, among which the most notable are the multidisciplinary UK Guidelines of 2004 and then 2014 (hereinafter referred to as the UK guidelines 2nd Edition), which were prepared according to principles of evidence-based medicine (*20*, *21*, *23*). Consensus guidelines from the Cystic Fibrosis Foundation for the diagnosis of CF recommend performing the ST according to validated protocols in order to ensure an acceptable quality of sweat collected as well as acceptable accuracy of the results (*12*).

## National guidelines for performance of the sweat test

A survey demonstrated the need for standardising the ST at the national level because of suboptimal practices at medical biochemical laboratories (MBLs) in Croatia (*19*). This survey therefore laid a solid foundation for developing the NGPST.

## Scope of the National guidelines

The NGPST are intended primarily for laboratory professionals and nurses involved in sweat testing, regardless of the method used, in order to facilitate standardisation of the ST across healthcare centres in Croatia. The guidelines are also intended for all healthcare professionals, as well as patients, parents/guardians and stakeholders interested in the performance and interpretation of the ST.

## Objective of the National guidelines

The NGPST are focused on standardisation of the ST across healthcare institutions in Croatia. Standardisation of the pre-analytical phase begins with ceasing the use of in-house equipment and materials for PI, which contradict the content and goals of the guidelines. Standardisation of the analytical phase means introducing an analytical instrument to determine sweat chloride using a validated method. Standardisation of the post-analytical phase, means generating a harmonised report of results, continuous user education and quality control. This process of ST standardisation may take time given its potential financial burden on local healthcare centres. Nevertheless, the NGPST may improve ST quality even in centres where introduction of commercial equipment and instruments is delayed. By following the NGPST, centres using in-house instruments may improve their performance of the ST until they can achieve full standardisation.

A standardised ST is necessary because it eliminates systematic error during all phases of testing, and it assays sweat chloride accurately in the target population.

## Working group for sweat test standardisation and development of the National guidelines

The Working Group for Sweat Test Standardisation (WG STS) was established in 2017 in cooperation with the Committee for Scientific and Professional Development on behalf of the Croatian Society of Medical Biochemistry and Laboratory Medicine (CSMBLM) and the Croatian Centre for Quality Assessment in Laboratory Medicine (CROQALM), an external provider of quality control for all MBLs in the country (*24*).

Since the performance of the sweat test is an interdisciplinary work, a CF health professional joined the WG STS. In most of those MBLs (12/13), ST has been performed using in-house method for PI and mercurimetric titration (MMT) according to the Schales and Schales micro-method to assay chloride concentration in sweat eluate. During guideline development, WG STS members communicated via email and in virtual meetings (teleconferences) to draft this document and to render final decisions that would be published as recommendations in the final version of the document.

The final version, was sent to the WG members for approval and was emailed to professional societies and associations for feedback and comments.

At the first teleconference in November 2017, it was decided that the NGPST should be based on the UK guidelines 2nd Edition, as they are the only existing evidence-based guidelines (*21*). Official permission to use the UK guidelines 2nd Edition in the NGPST was sought and granted by a contact person of the British Multidisciplinary Guideline Development Group, on the condition that instances be clearly marked where the UK guidelines were not applied because of specificities of performing the ST in Croatian healthcare institutions.

The NGPST were developed using questionnaires in survey conducted in 2018. The first was an update of a previously published survey covering all ST procedures (*19*). The second questionnaire examined the use of electrodes for PI and quality indicators, while the third investigated current power supply instruments. An additional questionnaire referred to the parts of the NGPST deviating from the UK guidelines 2nd Edition because of the ST equipment available for use in Croatian healthcare centres. The replies to all questionnaires are listed in Table 1 and Appendix 2.

**Table 1:** Overall results of the Working group survey

**Sweat test procedure**	**Number of MBLs and paediatric outpatient clinic (preanalytical phase)** **(Total number of centres = 9)**
**Preanalytical phase**	
In-house pilocarpine iontophoresis equipment and instruments	9
Flexor surface of the forearm as site of pilocarpine iontophoresis	9
Non-standard power supply	9
Two electrodes in the shape of bracelets	8
Pilocarpine chloride solution	8
Analytical balance sensitive to 0.0001 g	6
Filter paper for sweat collection	2
Gauze for sweat collection	7
Storage of filter paper or gauze	
Petri dish with a lid	3
plastic container	3
glass container	3
Sweat elution time	
at least 40 minutes	3
up to 1 hour	6
Storage of eluted sample	
2-8 °C	3
15-25 °C	4
no storage	2
**Analytical phase**	
Mercurimetic titration	9
In-house calibrator	8
In-house reagents only	7
Sample titration in duplicate	7
**Quality control**	
Internal quality control	5
External quality control	9

The NGPST contain concise recommendations for performing the ST. They have been taken from the UK guidelines 2nd Edition with permission, except where indicated by the phrase “recommendation independent of the UK guidelines 2nd Edition”. In these deviations, the WG STS arrived at consensus recommendations reflecting the particular ST equipment and procedures available in Croatia. More detailed information on the UK guidelines 2nd Edition can be found in the original document (*21*).

In order to harmonise the NGPST with the latest findings and recommendations about the ST appearing after the UK guidelines 2nd Edition, the WG members searched the MEDLINE database using the PubMed search engine to identify relevant publications from 01 January 2013 to 01 February 2021. Search terms included *cystic fibrosis, sweat test, sweat chloride, conductometry* and *biological variation.* The following publications were identified: national guidelines of professional societies (*12*, *20*), a review (*14*), and a cross-sectional study (*25*). In addition, the Google search engine was used to search for information available on the ST in Croatian (01 September – 01 December 2019).

Where applicable, the authors of the NGPST used the „Appraisal of Guidelines for Research and Evaluation“ (AGREE) tool (*26*).

The NGPST will be emailed to all WG members, professional societies and associations that took part in their development. An electronic version of the NGPST will be available in Croatian and English on the CSMBLM website.

## Resource implications of sweat test standardisation

According to an ECFS survey of 136 centres and laboratories in Europe, more than half offer the ST at prices ranging between 20 and 100 EUR (*27*). The Croatian Health Insurance Fund, a national compulsory health insurance reimburses the costs of the ST to contracted healthcare centres in the amount of 55.60 HRK or 7.50 EUR (*28*). Since performing the ST according to standardised procedures requires commercial systems and instruments that raise the cost of the test above this reimbursement level, MBLs can expect an additional financial burden. On the other hand, standardisation should ensure a high-quality diagnostic procedure, reducing the rate of false-negative or -positive results as well as the rate of quantity not sufficient (QNS) samples, ultimately reducing the overall healthcare system’s expenses related to CF diagnosis.

## Guidelines contents

### Indications for sweat testing

(recommendations independent of the UK guidelines 2nd Edition)

A primary care physician should refer a patient with suspected CF to a CF specialist for further diagnostic work-up.

Once a national CF newborn screening is introduced, newborns with a positive screening result should be referred to a CF centre.

### Informing the patients and parents/guardians about the sweat test

Before testing, the patients and parents/guardians should be informed about the purpose of the ST and about the patient’s indication for the test.

MBL should ensure pre-test information about the sweat test (recommendations independent of the UK guidelines 2nd Edition).

Pre-test information should give insight into the purpose of the test, patient preparation, the test procedures, associated risks, and contacts details about testing.

An example of an ST information leaflet can be found in Appendix 3 and can be modified according to the MBL or the clinical needs (recommendations independent of the UK guidelines 2nd Edition).

### Patient’s suitability for the sweat test

ST is recommended for a term infant (newborn) who is two weeks old and weighs more than 2 kg at the time of testing, and who is normally hydrated without significant systemic disease.

ST is not recommended before the first 7 days of life, especially the first 48 hours, as tests during this period show unacceptably high rates of false-positive results or PI failure.

ST should be delayed in subject who are oedematous or who are receiving topiramate or 9-alpha fludrocortisone.

Testing should also be delayed in subjects who show dehydration, malnutrition, unstable clinical condition or eczema at the site of sweat stimulation.

ST can be performed in subjects on Flucloxacillin therapy.

ST is not recommended in subjects receiving oxygen by an open delivery system, such as a headbox. This recommendation does not apply to a child receiving oxygen through a nasal prong or face mask.

### The newborn screening for cystic fibrosis and sweat test

The National Programme for Rare Diseases 2015-2020, issued by the Ministry of Health of the Republic of Croatia, includes CF and recognises newborn screening for CF as a practice in some European countries (*13*). However, it is not part of the newborn screening programme in Croatia.

The following recommendations are necessary in light of the importance of the ST in newborn screening for CF:

A positive result of newborn screening for CF should be confirmed by an ST involving PI and measurement of chloride concentration in sweat.

The ST should be performed by a laboratory/outpatient clinic staff experienced in testing infants younger than 3 months old.

## Sweat test procedures

### Sites for the pilocarpine iontophoresis

The flexor surface of either forearm is the recommended site for PI.

Other acceptable sites are the upper arm or thigh if both arms are eczematous or otherwise unsuitable for sweat stimulation and collection.

Using cleaning solutions containing chloride is not recommended.

Performing the ST on only one arm is sufficient except in the event of sample contamination, QNS sample or other abnormality.

### Pilocarpine iontophoresis

#### Recommendations for in-house PI

The European Pharmacopoeia should be followed when preparing solutions for in-house PI (recommendation independent of the UK guidelines 2nd Edition).

The power supply must be battery-powered and should include a safety cut-out that limits the amount of current to a maximum of 5 mA.

The two electrodes should be made of stainless steel or copper, with a shape and size that allow them to be fastened securely to the subject’s forearm without pain or excessive pressure (recommendation independent of the UK guidelines 2nd Edition).

The electrodes should be regularly inspected and cleaned.

The recommended electrode size is 3.75 x 3.75 cm (recommendation independent of the UK guidelines 2nd Edition).

Pilocarpine nitrate solution (2-5 g/L) should be used to stimulate sweat.

The chloride-free filter paper or gauze for sweat stimulation should be larger than the electrodes used for PI (recommendation independent of the UK guidelines 2nd Edition).

Electrodes should be assembled with filter paper or gauze soaked in pilocarpine nitrate solution and then fixed to the flexor surface of the forearm in a way that prevents electrode movement and bridging with pilocarpine solution during sweat stimulation (recommendation independent of the UK guidelines 2nd Edition).

A maximum current of 4 mA should be applied for at least 3 minutes but no more than 5 minutes.

Chloride free filter paper or gauze for sweat collection should be larger than the stimulated area: for example, gauze or filter paper measuring 5 x 5 cm should be used for electrodes measuring 3.75 x 3.75 cm (recommendation independent of the UK guidelines 2nd Edition).

Before PI, the filter paper or gauze should be placed in labelled, tightly closed containers and weighed using an analytical balance sensitive to at least 0.0001 g.

Every container with a sweat sample should be labelled with a patient’s barcode or permanent ink (recommendation independent of the UK guidelines 2nd Edition).

Pre-weighed filter paper or gauze for sweat collection should be placed as soon as possible at the site of PI and fixed in a way that prevents evaporation and contamination of sweat during collection.

Sweat collection should take 30 minutes.

After sweat collection, the filter paper or gauze should be returned to the same tightly closed and container as for weighing, then delivered to the laboratory if the PI is performed in outpatient clinic (recommendation independent of the UK guidelines 2nd Edition).

The filter paper or gauze with collected sweat should be reweighed before chloride analysis (recommendation independent of the UK guidelines 2nd Edition).

Sweat samples collected from different sites should not be mixed and analysed (recommendation independent of the UK guidelines 2nd Edition).

The user must verify the instrument, equipment and materials used for in-house PI (recommendation independent of the UK guidelines 2nd Edition).

#### Recommendations for PI using commercial equipment

The commercial instrument must be battery-powered with an automatic safety cut-out.

Pilocarpine gel discs containing pilocarpine nitrate at a concentration of 2-5 g/L should be used to stimulate sweating.

Pilocarpine gel discs should not be used if any damage is noticed (recommendation independent of the UK guidelines 2nd Edition).

A commercial system including disposable collectors for sweat collection is recommended.

Parts of commercial kits should not be combined with in-house instruments, equipment or materials for sweat stimulation or collection.

### Sample stability and preparation for analysis

Sweat collected with a commercial kit can be stored for a maximum of 3 days at 2-8 °C in a tightly closed container.

Sweat collected on filter paper or gauze can be kept at 2-8 °C for a maximum of 3 days in a tightly closed container.

The weight of collected sweat should be consistent with a request of a minimum sweat secretion rate of 1 g/m^2^/min.

Each laboratory, depending on the equipment used, should define a minimum weight of collected sweat that is acceptable for the analytical phase (Appendix 4: „Calculation of the minimum acceptable sweat weight“) (recommendation independent of the UK guidelines 2nd Edition).

Collected sweat weighing less than the defined minimum acceptable weight should not be analysed.

Sweat elution longer than 1 hour is not recommended (recommendation independent of the UK guidelines 2nd Edition).

Sample collected onto filter paper or gauze should be mixed well before analysis.

### Sweat analysis

Chloride is the analyte of choice when analysing sweat for CF diagnosis.

Measurement of osmolality or concentrations of sodium or potassium in sweat is not recommended.

Quantitative colorimetry, coulometry and ion-selective electrodes (ISE) are the methods recommended for sweat chloride analysis.

Conductivity is not acceptable as a reference method for CF diagnosis.

In children younger than 6 months, an ST involving sweat chloride measurement should be performed if a conductivity test gives a negative result.

In case of a positive or borderline conductivity result, an ST measuring chloride concentration should be performed in children older than 6 months and adults.

#### Recommendation for chloride analysis

(recommendations independent of the UK guidelines 2nd Edition)

Mercurimetric titration according to the Schales and Schales micro-method is an acceptable analytical technique for chloride analysis in sweat or eluted sweat only if the technique has been validated in that matrix.

Mercury nitrate solution used in the MMT must be safely removed from MBLs according to laboratory waste management standards.

#### Conductivity method

The chloride concentrations in sweat determined using the Nanoduct or any other instrument based on conductivity (e.g. Sweat Check Analyser, ELITechGroup, USA) are generally higher than the chloride concentrations determined using analytical methods that measure only chloride, such as coulometry. The authors of the British and Australasian guidelines for the performance of ST consider the conductivity method unacceptable for CF diagnosis (*20*, *21*). According to the guidelines for the performance of the ST of the American Clinical and Laboratory Standard Institute, a NaCl equivalent of ≥ 50 mmol/L obtained by conductivity should be confirmed by measurement of chloride concentration in sweat (*23*). Unpublished data, indicated that the Nanoduct was used in eight of nine paediatric units and one of nine MBLs across five general hospitals, one county hospital, two specialty hospitals and one clinical hospital center covering most counties in Croatia.

## Quality control

Contaminated or evaporated sweat samples should not be analysed (see section on PI above).

The analytical method must show a linear response over the range of chloride concentrations in the sweat of healthy and CF subjects.

The procedures of the ST must be documented in the quality management system and in accordance with national professional standards (recommendation independent of the UK guidelines 2nd Edition).

Internal quality control (IQC) must be applied to every batch of samples.

In-house IQC samples can be used (recommendation independent of the UK guidelines 2nd Edition).

For every batch of samples, IQC should be conducted at three concentrations: normal level, < 30 mmol/L; borderline level, 30-59 mmol/L; and abnormal level, ≥ 60 mmol/L (recommendation independent of the UK guidelines 2nd Edition).

Methods for determining chloride (but not conductivity) should have between-batch imprecision lower than 5% at concentrations of 40-50 mmol/L.

Conductivity method should have between-batch imprecision lower than 2% at a concentration of 50 mmol/L.

The laboratory must participate in an external quality assessment (EQA) scheme.

Records of results of internal and external quality control must be kept as part of medical laboratory documentation in accordance with national regulation (recommendation independent of the UK guidelines 2nd Edition).

Since 2015, the CROQALM has provided national EQA for the ST analytical phase based on a separate module in three rounds annually (one sample per round) (*24*). All MBLs in Croatia that perform the ST and report results participate in this national EQA. Between 2015 and 2020, the control material was in-house aqueous NaCl solution at a chloride concentration range of 20-100 mmol/L. Since 2021, a commercial quality control system has been in use. The report contains graphical and tabular representations, statistical analysis of the results according to the Tukey method, as well as an interpretation of the reported chloride concentration (*29*).

Non-physiological results (chloride concentration > 150 mmol/L or conductivity > 170 mmol/L) should be investigated for errors.

The laboratory or outpatient clinic should monitor the rates of QNS samples due to insufficient weight or volume of sweat collected. The QNS should not exceed 10% of the tested population, excluding repeat sampling and children younger than 6 months. There should be a target of less than 5% in children older than 6 months. Among children younger than 6 months, the rate of failed sweat collection should not exceed 20% in the tested population. The QNS rate should be expressed as a percentage (%) of total tests and as a percentage (%) per operator, and records of the QNS rate and the associated reports should be maintained. Appendix 5 lists quality indicators for the performance of the ST.

Testing should be repeated if the result is inconsistent with the subject’s clinical condition and/or with results of genetic testing.

Appendix 6 lists causes and conditions that can contribute to false positive and false negative ST results (*20*).

Results should be reviewed with clinicians, in particular repeat sweat collections and positive results.

## Reference values and interpretation of results

### Chloride concentration by colorimetry, coulometry, ion selective elektrodes (recommendation independent of the UK guidelines 2nd Edition) (*12*)

Normal level ≤ 29 mmol/L

Borderline level 30-59 mmol/L

Elevated level ≥ 60 mmol/L

### NaCl equivalents by the conductivity method

Normal level < 50 mmol/L

Borderline level 50-90 mmol/L

Elevated level > 90 mmol/L

### Biological variation and reference change value

(recommendation independent of the UK guidelines 2nd Edition)

The laboratory may use data from the biological variation database that are appropriate for the tested population.

A laboratory that employs a standardised procedure for PI and sweat chloride analysis may use reference change value in order to evaluate the clinical significance of testing results (*25*).

## The sweat test report

The report format should include full information for patient identification, date and time of the test and report, the analyte measured, the analyte result, analytical method used, reference ranges and interpretation of the results, as well as a reason if no result is reported.

The format of reports from MBLs should be in line with the National Recommendation for Post-analytical Laboratory Work (*30*) (recommendation independent of the UK guidelines 2nd Edition).

The weight or volume of the collected sweat should be stated in the laboratory report (*20*) (recommendation independent of the UK guidelines 2nd Edition).

## Responsibility for training and testing

Sweat collection and analysis should be performed only by qualified and skilled operators.

All levels of operators training should be documented.

The appropriate procedures for training staff and assessing their ST skill should be defined.

Trained operators should perform a minimum of 10 tests per year in order to maintain testing quality (*20*) (recommendation independent of the UK guidelines 2nd Edition).

An individual holding a master’s degree in medical biochemistry, a specialist in medical biochemistry and laboratory medicine, a medical doctor or a specialist in laboratory medicine should be responsible for training and evaluating staff who perform ST in the MBL as well as for supervising the entire workflow, including quality assessment and revision of quality indicators, reporting of results and discussions with specialists. Every phase of the ST in the MBL should be controlled by a qualified specialist, including analysis of unexpected results and implementation of corrective actions (recommendation independent of the UK guidelines 2nd Edition).

If the ST is partially or completely performed outside the MBL, an individual holding a master’s degree in medical biochemistry, a specialist in medical biochemistry and laboratory medicine, a medical doctor or a specialist in laboratory medicine should evaluate the frequency of quality control assessment (including revision of quality indicators) and unexpected results as well as implementation of corrective actions (recommendation independent of the UK guidelines 2nd Edition).

The doctor or nurse who supervises staff performing ST fully or in part outside the MBL should keep records on personnel training, evaluation and competence. The supervisor should verify that the ST is performed in accordance with written instructions, and that test results and quality control assessments (including rates of QNS and failed tests per operator) are fully documented (recommendation independent of the UK guidelines 2nd Edition).

## Supervision of the implementation of the guidelines in medical biochemistry laboratories

The NGPST developed through the joint work of the CSMBLM and CSPGHN will be evaluated by the Committee for Professional Concerns of the Croatian Chamber of Medical Biochemists and it will subject of the public disscusion by the CSMBLM members, prior to publication.

## Appendix 2 Overall results of the Working group survey on the preanalytical and analytical phases of the sweat test

### Preanalytical phase

In 2018 the stimulation of sweating using in-house PI equipment and instruments was a routine procedure in nine healthcare centres, including six MBLs and three paediatric outpatient clinic at secondary and tertiary healthcare centres. In one centre, the PI was part of a commercial system that relies on the conductivity method. Data from this MBL was not included in the following presentation of survey results.

In all centres, sweat stimulation and collection were performed on the flexor surface of the left or right forearm. They used the same instrument for sweat stimulation (JOS Instruments, Croatia), a device that was not battery-powered and that lacks a safety cut-out. In eight of nine centres, PI was conducted using two electrodes (JOS Instruments) in the shape of bracelets with a width of 8 mm and an adjustable length up to 30 cm. Eight of the nine centres stimulated sweating using in-house pilocarpine chloride solution. Six of nine MBLs used an analytical balance sensitive to 0.0001 g. Two of nine centres collected sweat using filter paper, but they did not report using recommended chloride-free filter paper; the remaining seven centres collected sweat using gauze. After sweat collection, three of the nine centres stored the filter paper or gauze in Petri dishes with a lid, three centres stored it in plastic containers, and the remaining three stored it in glass containers. Three of nine centres eluted sweat with distilled water for a minimum of 40 minutes, while the remaining six MBLs eluted it for up to 1 hour. Eluates were stored at 2-8 °C in three MBLs, 15-25 °C in four MBLs, or not at all in two MBLs. The queastionnarie did not ask about how long eluted samples were stored. At the end of 2018, only three of nine centres recorded undesirable events during PI. Eight of nine centres considered inadequate the instrument they used for sweat stimulation.

### Analytical phase

All MBLs analysed chloride concentrations in sweat eluate using the MMT according to the Schales and Schales micromethod. Eight of nine laboratories used in-house calibrator, while one used a commercial calibrator. Two of nine MBLs used a combination of commercial and in-house reagents for the MMT, while the remaining seven used only in-house reagents. Seven of the nine MBLs performed titration in duplicate.

Five of nine laboratories performed IQC, but all nine participated in the EQA conducted by the CROQALM during three cycles *per* year. Two of the nine laboratories also participated in the ST module of the Randox International Quality Assesment Scheme.

## Appendix 3 An example of the sweat test information leaflet

This information leaflet is intended for patients and parents/guardians of children who have been referred for a sweat test (ST).

What is the sweat test?

The ST is a simple, non-invasive procedure performed in the following phases: stimulation of sweating, collection of sweat and analysis of chloride in the sweat. The testing is painless but may produce a tingling sensation at the site of sweat stimulation.

Why is the sweat test performed?

Children who have frequent diarrhoea or respiratory infection, who fail to thrive or who have other symptoms of cystic fibrosis (CF) may be referred for an ST. High chloride concentration in sweat can indicate CF, but the final diagnosis must be made by a CF specialist after taking into account the child’s symptoms, family medical history and the results of other diagnostic tests.

Where and when is the sweat test performed?

The ST is generally performed in the laboratory, paediatrician unit or outpatient clinic. Tests can be scheduled by phone or email.

What is good to know before having a sweat test?

Success of the ST depends strongly on the child’s condition and on parents’/ guardians’ familiarity with the test. On the day before testing, children should consume 0.5-2.0 L of liquid (depending on age). On the day of testing, the child should be well hydrated with no fever, oedema or skin rash on either forearm. No cream or lotion should be applied to the child’s hands on the day of testing. The child should consume a normal meal before the testing.

How is the sweat test performed?

The test is performed in the following phases:

Phase one – stimulation of sweating

The flexor surface of the child’s forearm is cleaned with distilled water and dried. Gauze or filter paper soaked in a specific solution is applied to the skin with two electrodes on the top, then fixed to the forearm. The electrodes are connected to a power supply, which delivers a low current to make the skin sweat. This takes less than 5 minutes, during which the child may experience a slight burning sensation in the stimulated area.

Phase two – collection of sweat

After sweat stimulation, the gauze or filter paper is removed by hand, and the skin is cleaned with water. The stimulated area may be red. Next, filter paper or gauze for sweat collection is fixed to the stimulated area, and sweat is collected for 30 minutes, during which the child can move, play, eat and drink. Salty snacks should be avoided. If the subject is an infant, he or she should be kept warm. When sweat collection is over, the filter paper or gauze is removed from the child’s forehand.

Phase three – analytical phase

The chloride concentration in the collected sweat is measured in a laboratory.

How long does it take to get the sweat test result?

Generally, the result is reported in 24 hours.

Information about test results

The report is delivered electronically to the doctor who ordered the ST.

Additional information

Any additional information can be delivered by telephone or email. The test result is interpreted by the CF specialist who ordered the ST.

## Appendix 4 Calculation of the minimum acceptable sweat weight

The minimum weight of collected sweat should be calculated according to the following equation, assuming a sweat secretion rate of 1 g/m^2^/min:

m – weight of collected sweat (mg)

t – collection time (minutes)

A – surface area of the gauze or filter paper used to collect sweat (cm^2^)

An example of the calculation:

t = 30 min

A = 25 cm^2^ (e.g. gauze or filter paper measuring 5 x 5 cm)

m (mg) = 25 (cm^2^) x 30 (min) / 10 = 75

## Appendix 5 List of quality indicators for performance of the sweat test

Results of internal quality control:

between-batch imprecision should be < 5% at chloride concentrations of 40-50 mmol/L

between-batch imprecision should be < 2% at chloride concentrations of 50 mmol/L (conductivity method)

Results of the EQA

Percentage of QNS samples of sweat in the tested population during periodic analysis:

< 5% in children older than 6 months

< 20% in children younger than 6 months

< 10% in target population

Percentage of QNS samples *per* operator

## Appendix 6 Causes and conditions that can contribute to false positive and false negative the sweat test results

Patient’s nutritional and hydration state

Skin contamination with a chloride-containing agent

Insufficient fluid removal following PI

Failure to stimulate sweating

Sweat evaporation during collection, transport or weighing

Inappropriate selection of analytical method

Improper performance of the selected method

Lack of skill in performing the selected method
